# Cutaneous metastasis reveling lung cancer

**DOI:** 10.11604/pamj.2015.20.19.5883

**Published:** 2015-01-07

**Authors:** Fatima Zahra Elfatoiki, Fouzia Hali

**Affiliations:** 1 Department of Dermatology, Ibn Rochd UHC of Casablanca, Morocco

**Keywords:** Lung, skin nodules, adenocarcinoma

## Image in medicine

A 52-year old man, chronic smoker for 20 years, presented at our department with skin nodule, firm, painful and ulcerated, localized in the trunk, rapidly grown for period of three months. Histological and immunohistochimical examination confirmed the lesion to be metastasis of lung adenocarcinoma. The patient underwent a CT scan of the abdomen and thorax, which revealed primytumor of the left lower lobe T4N2M+ with infiltration of the deep fascial and muscular planes by theneoplasm associated with hepatic metastasis and vertebral metastasis T9-T11. The patient died after 1 month.

**Figure 1:**
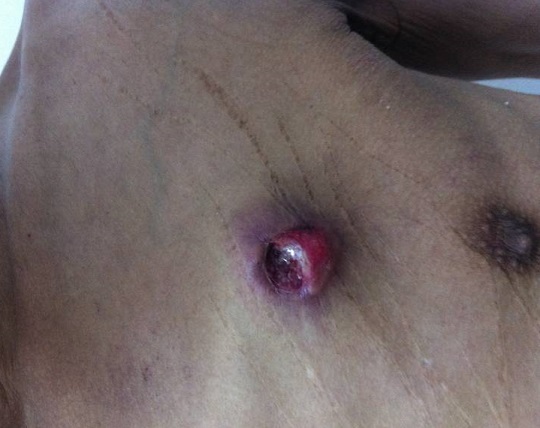
Ulcerated nodule of the trunk

